# Reversal of metabolic disorders by pharmacological activation of bile acid receptors TGR5 and FXR

**DOI:** 10.1016/j.molmet.2018.01.005

**Published:** 2018-01-11

**Authors:** Kavita Jadhav, Yang Xu, Yanyong Xu, Yuanyuan Li, Jiesi Xu, Yingdong Zhu, Luciano Adorini, Yoon Kwang Lee, Takhar Kasumov, Liya Yin, Yanqiao Zhang

**Affiliations:** 1 Department of Integrative Medical Sciences, Northeast Ohio Medical University, Rootstown, OH, 44272, USA; 2 State Key Laboratory of Molecular Developmental Biology, Institute of Genetics and Developmental Biology, Chinese Academy of Sciences, Beijing, 100101, China; 3 Intercept Pharmaceuticals, New York, NY, 10014, USA; 4 Department of Pharmaceutical Sciences, Northeast Ohio Medical University, Rootstown, OH, 44272, USA

**Keywords:** Farnesoid X receptor, TGR5, Atherosclerosis, Obesity, NAFLD, α-Sma, alpha smooth muscle actin (*α-Sma*), BA, bile acid, BAT, brown adipose tissue, CEBPα, CCAAT/enhancer-binding protein α, Col1α1, collagen type 1 α1, DIO, diet-induced obesity, FXR, farnesoid X receptor, GLP-1, glucagon-like peptide-1, G6pc, glucose 6-phosphatase, HFD, high fat diet, IL-1β, interleukin 1β, NAFLD, non-alcoholic fatty liver disease, NASH, non-alcoholic steatohepatitis, NS, not significant, Pck1, phosphoenoylpyruvate carboxylase 1, PPARγ, peroxisome proliferation-activated receptor γ, SHP, small heterodimer partner, TG, triglyceride, TGR5, G protein coupled bile acid receptor (GPBAP), Timp1, tissue inhibitor of metalloproteinase 1, TNFα, tumor necrosis factor α, Tgfβ, transforming growth factor β

## Abstract

**Objectives:**

Activation of the bile acid (BA) receptors farnesoid X receptor (FXR) or G protein-coupled bile acid receptor (GPBAR1; TGR5) improves metabolic homeostasis. In this study, we aim to determine the impact of pharmacological activation of bile acid receptors by INT-767 on reversal of diet-induced metabolic disorders, and the relative contribution of FXR vs. TGR5 to INT-767's effects on metabolic parameters.

**Methods:**

Wild-type (WT), *Tgr5*^−/−^, *Fxr*^−/−^, *Apoe*^−/−^ and *Shp*^−/−^ mice were used to investigate whether and how BA receptor activation by INT-767, a semisynthetic agonist for both FXR and TGR5, could reverse diet-induced metabolic disorders.

**Results:**

INT-767 reversed HFD-induced obesity dependent on activation of both TGR5 and FXR and also reversed the development of atherosclerosis and non-alcoholic fatty liver disease (NAFLD). Mechanistically, INT-767 improved hypercholesterolemia by activation of FXR and induced thermogenic genes via activation of TGR5 and/or FXR. Furthermore, INT-767 inhibited several lipogenic genes and *de novo* lipogenesis in the liver via activation of FXR. We identified peroxisome proliferation-activated receptor γ (PPARγ) and CCAAT/enhancer-binding protein α (CEBPα) as novel FXR-regulated genes. FXR inhibited PPARγ expression by inducing small heterodimer partner (SHP) whereas the inhibition of CEBPα by FXR was SHP-independent.

**Conclusions:**

BA receptor activation can reverse obesity, NAFLD, and atherosclerosis by specific activation of FXR or TGR5. Our data suggest that, compared to activation of FXR or TGR5 only, dual activation of both FXR and TGR5 is a more attractive strategy for treatment of common metabolic disorders.

## Highlights

• Bile acid receptor activation improves lipid homeostasis primarily via activation of FXR.
• Bile acid receptor activation reverses DIO and induces energy expenditure.
• Bile acid receptor activation reverses diet-induced NAFLD and atherosclerosis.
• Bile acid receptor activation inhibits hepatic lipogenesis via activation of FXR.
• Activation of FXR inhibits PPARγ dependent on SHP and CEBPα independent of SHP.

## 1 Introduction

Non-alcoholic fatty liver disease (NAFLD) is a spectrum of liver diseases ranging from simple steatosis to non-alcoholic steatohepatitis (NASH), which may further progress to liver cirrhosis and liver carcinoma [1], [2]. While hepatic steatosis is considered a benign condition, a “two-hit” theory has been proposed to explain the pathogenesis of NAFLD [3], [4]. The first hit consists of abnormal accumulation of triglycerides (TG) in the liver. If steatotic liver suffers a second hit, including inflammatory mediators, reactive oxygen species, and activation of stellate cells, it may progress to NASH. NAFLD is often associated with obesity and diabetes and is also an independent risk factor for cardiovascular disease [5], [6], [7], [8].

Bile acids (BAs) are amphipathic molecules. Primary BAs are synthesized from cholesterol exclusively in the liver whereas secondary BAs are synthesized in the intestine. BAs help absorption of dietary lipids from the intestine and are endogenous ligands for the nuclear receptor farnesoid X receptor (FXR) and the G protein-coupled receptor bile acid receptor TGR5 (also called GPBAR1). Chenodeoxycholic acid and lithocholic acid are the most potent endogenous activators of FXR and TGR5, respectively, while cholic acid is the least potent activator of both receptors [1]. Nonetheless, BA can also activate other nuclear receptors or pathways [9]. The two BA receptors have overlapping but also differing tissue and cell distributions. FXR is mostly expressed in the liver (hepatocytes only), intestine, kidney, and adrenal glands [10] while TGR5 has a wider expression pattern including gallbladder, small intestine, brown adipose tissue (BAT), spleen, and macrophages [11]. In the liver, TGR5 is only expressed in sinusoidal endothelial cells and Kupffer cells, the resident macrophages of the liver, but not in hepatocytes [12].

Many lines of evidence have clearly demonstrated that activation of FXR improves lipid and glucose homeostasis and inhibits the development of NAFLD and atherosclerosis [1], [13]. Activation of TGR5 improves glucose and energy homeostasis and inhibits atherogenesis by inducing glucagon-like peptide-1 (GLP-1) secretion in the intestine and thermogenic genes in BAT and skeletal muscle [14], [15] and suppressing macrophage inflammation [16]. However, long-term activation of FXR can also induce obesity by reducing the BA pool size [17]. In addition, activation of TGR5 appears to have a limited impact on lipid homeostasis [16]. Thus, development of dual agonist(s) for both FXR and TGR5 appears to be a more attractive strategy for treatment of common metabolic disorders.

INT-767 is a semisynthetic, potent, and specific agonist for both FXR and TGR5, with EC50 values of 30 nM and 630 nM, respectively [18]. INT-767 reduces monocyte infiltration and inhibits NASH development in *db*/*db* mice [19] and suppresses the development of atherosclerosis in *Apoe*^−/−^ mice [20]. So far, it is unclear whether INT-767 treatment can reverse diet-induced metabolic disorders or atherosclerosis and how INT-767 treatment improves metabolic disorders. In this report, we utilized wild-type (WT) mice, *Fxr*^−/−^ mice, and *Tgr5*^−/−^ mice to elucidate the relative importance of FXR versus TGR5 in INT-767-regulated metabolic homeostasis. We also utilized small heterodimer partner (*Shp*) knockout (*Shp*^−/−^) mice to address how activation of FXR inhibits lipogenic genes. In addition, we investigated whether INT-767 could reverse atherosclerosis in *Apoe*^−/−^ mice. Our data indicate that INT-767 reverses obesity, hypercholesterolemia, NAFLD, and atherosclerosis by activation of FXR and/or TGR5. Dual activation of FXR and TGR5 is an attractive strategy for treatment of common metabolic disorders.

## 2 Materials and methods

### 2.1 Mice, diets, and agonists

*Fxr*^−/−^ mice (originally from the Jackson Laboratory, ME) [21], *Tgr5*^−/−^ mice (originally from Merck Research Laboratories; Kenilworth, NJ) [22], and *Shp*^−/−^ mice [23] have been described previously. The control wild-type mice were the littermates of knockout mice. All the mice were on a C57BL/6 background. Mice were fed a chow diet, high fat diet (60% kcal from fat; Research Diets, D12492) or Western diet (Envigo, TD.88137), or gavaged with either vehicle (0.5% carboxymethyl cellulose, Sigma), INT-767 (30 mg/kg, once a day), or GW4064 (30 mg/kg, twice a day) for up to 12 weeks. INT-767 and GW4064 were provided by Intercept Pharmaceuticals (New York, NY) and GlaxoSmithCline (North Carolina), respectively. Unless otherwise stated, all mice were fasted for 5–6 h prior to euthanization. All the animal studies were approved by the Institutional Animal Care and Use Committee at Northeast Ohio Medical University and were consistent with the National Institutes of Health guide for the care and use of Laboratory animals.

### 2.2 Real-time PCR

RNA was isolated using TRIzol Reagent (Life Technologies). mRNA levels were determined by quantitative reverse-transcription polymerase chain reaction (qRT-PCR) on a 7500 real-time PCR machine from Applied Biosystems (Foster City, CA) by using SYBR Green Supermix (Roche, Indianapolis, IN). Results were calculated using *Ct* values and normalized to *36B4* mRNA level.

### 2.3 Transfections and cell culture

HepG2 cells were purchased from ATCC (Virginia) and cultured in DMEM plus 10% FBS. pGL3-PPARγ2 (−2.5 kb) plasmid DNA and plasmids expressing HNF4α, SHP, or β-galactosidase (β-gal) were transfected into HepG2 cells. After 36 h, luciferase activity was determined and normalized to β-gal activity.

### 2.4 Western blot and immunostaining assays

Western blot assays were performed using Smad2 antibodies (phospho- or total; Cat #s 8828 and 5678) and Caspase 3 antibodies (cleaved and total; Cat #s 9661 and 9662) were purchased from Cell Signaling Technology (Boston, MA, USA). Tubulin antibody was purchased from Abcam (Cambridge, MA, USA). Immunostaining was performed using an ABC-HRP kit from Vector Laboratories (Burlingame, CA, USA. Cat # PK-4001) and primary antibodies from Abcam, including F4/80 (Cat # ab6640), VCAM-1 (Cat # ab134047), MCP-1 (Cat # ab25124) and 4-HNE (Cat # ab46545). TUNEL kit was purchased from Abcam (Cat # ab206386).

### 2.5 Lipid and lipoprotein analysis

Approximately 100 mg liver tissue was homogenized in methanol, and lipids were extracted in chloroform/methanol (2:1 v/v) as described [24]. Hepatic triglyceride (TG) and cholesterol levels were then quantified using Infinity reagents from Thermo Scientific (Waltham, MA, USA). Plasma TG, cholesterol, and glucose levels were also determined using Infinity reagents. Plasma lipoprotein profile was analyzed by fast protein liquid chromatography (FPLC) as described previously [25], [26].

### 2.6 De novo lipogenesis

Male mice were injected i.p. with ^2^H_2_O. After 4 h, *de novo* lipogenesis was carried out by GC mass spectrometry as described previously [25], [27].

### 2.7 Glucose tolerance test and insulin tolerance test

Male mice were fasted for 6 h. Glucose tolerance test and insulin tolerance test were performed by injecting mice i.p. with 2 g/kg glucose or 0.85 units/kg insulin, respectively, as described previously [28].

### 2.8 VLDL secretion

Male mice were i.v. injected with Tyloxapol (500 mg/kg). Blood was taken at indicated time points to measure plasma triglyceride levels, as described [25].

### 2.9 Atherosclerotic lesions

The aortas and aortic roots were isolated. Sectioned aortic roots or *en face* aortas were stained with Oil Red O and the atherosclerotic lesion size determined as described previously [26], [29].

### 2.10 Liver histology and biochemical assays

Livers were fixed in 4% formalin and then embedded in OCT or paraffin. Neutral lipids were stained with Oil red O [25], and liver collagens were stained with picrosirius red. Hepatic MDA levels were measured using a TBARS assay kit from Cell Biolabs (San Diego, CA. Cat # STA-330). Hepatic hydroxyproline levels were measured using a kit from Cell Biolabs (Cat # STA-675).

### 2.11 Energy expenditure

Mice fed a Western diet were put in the Comprehensive Lab Animal Monitoring System (CLAMS). Oxygen consumption, CO_2_ production, and heat production were determined as described [30], [31]. Body fat was measured by Echo-MRI (EchoMRI, LLC, TX).

### 2.12 Statistical analysis

Statistical significance was analyzed using unpaired Student *t* test or ANOVA (GraphPad Prism, CA). All values are expressed as mean ± SEM. Differences were considered statistically significant at *P* < 0.05.

## 3 Results

### 3.1 INT-767 regulates lipid metabolism via activation of FXR in chow-fed mice

**Figure 1:**
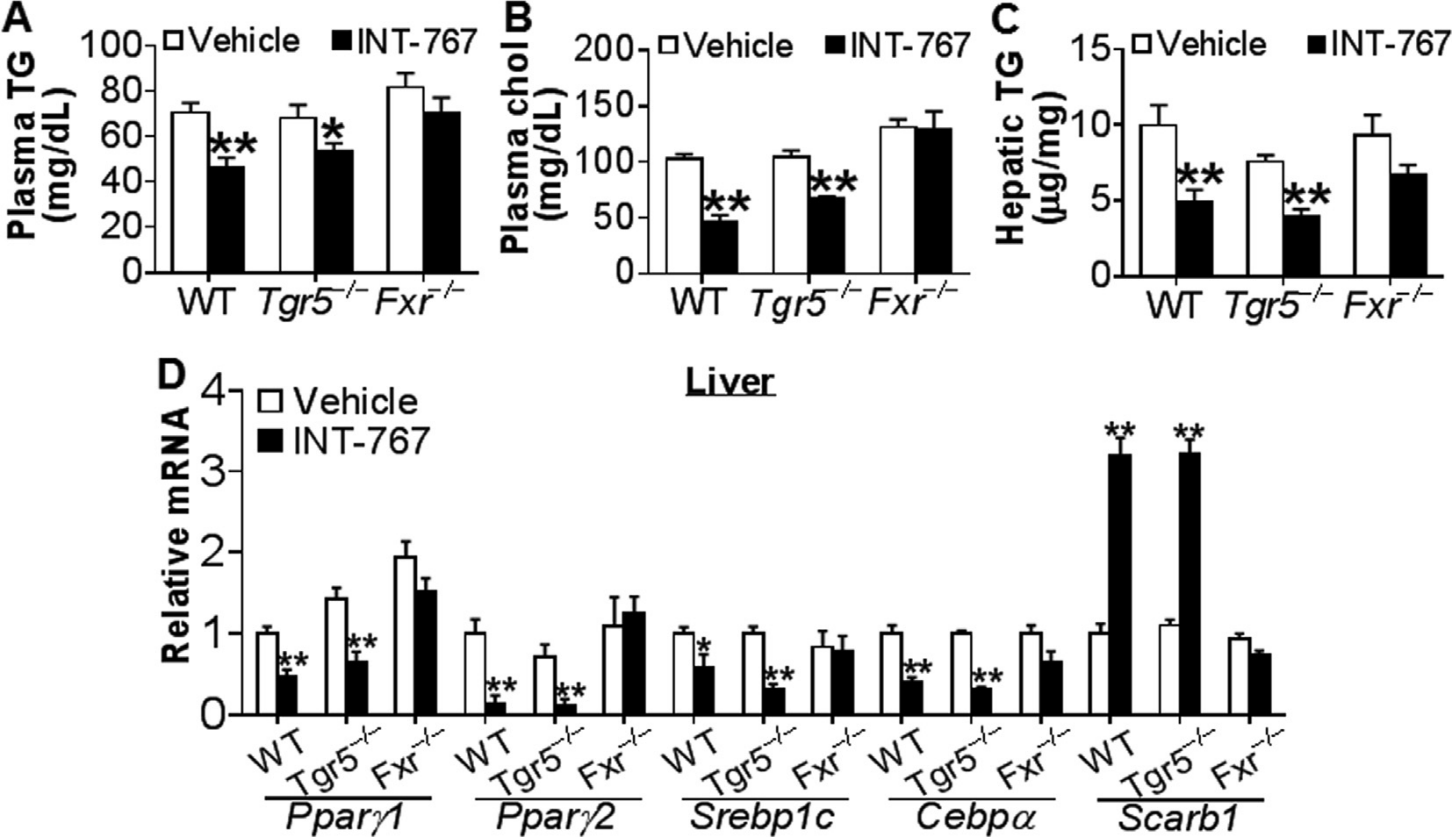
**INT-767 regulates lipid metabolism by activation of FXR in chow-fed mice**. Chow-fed male wild-type (WT) mice, *Tgr5*^−/−^ mice or *Fxr*^−/−^ mice were gavaged with either vehicle or INT-767 once a day for 7 days. (A) Plasma TG levels. (B) Plasma cholesterol levels. (C) Hepatic TG levels. (D) Hepatic mRNA levels. *P < 0.05, **P < 0.01.

INT-767 is a semi-synthetic dual agonist for both FXR and TGR5 [18]. To determine whether INT-767 regulated lipid metabolism via activation of FXR and/or TGR5, initially we gavaged chow-fed wild-type (WT), *Fxr*^−/−^ and *Tgr5*^−/−^ mice with either vehicle or INT-767 for 7 days. INT-767 treatment significantly reduced plasma TG, plasma cholesterol, and hepatic TG levels in both WT mice and *Tgr5*^−/−^ mice but not in *Fxr*^−/−^ mice (Figure 1A–C). There was no change in hepatic cholesterol levels (data not shown). Consistent with these data, INT-767 inhibited hepatic peroxisome proliferation-activated receptor γ1 (*Pparγ1*), *Pparγ2*, sterol regulatory element-binding protein 1c (*Srebp1c*), and CCAAT/enhancer binding protein α (*Cebpα*) but induced scavenger receptor group B type I (*Scarb1*/*SR-BI*) in both WT mice and *Tgr5*^−/−^ mice but not in *Fxr*^−/−^ mice (Figure 1D). PPARγ1 [32], PPARγ2 [33], SREBP1C [34], and CEBPα [35] are all known to promote lipogenesis. SR-BI is the HDL receptor in the liver for plasma HDL uptake [36] and is known to be induced by FXR activation [37]. These data demonstrate that INT-767 improves lipid homeostasis via activation of FXR in chow-fed mice.

### 3.2 INT-767 treatment reverses high fat diet-induced metabolic disorders via activation of FXR and/or TGR5

**Figure 2:**
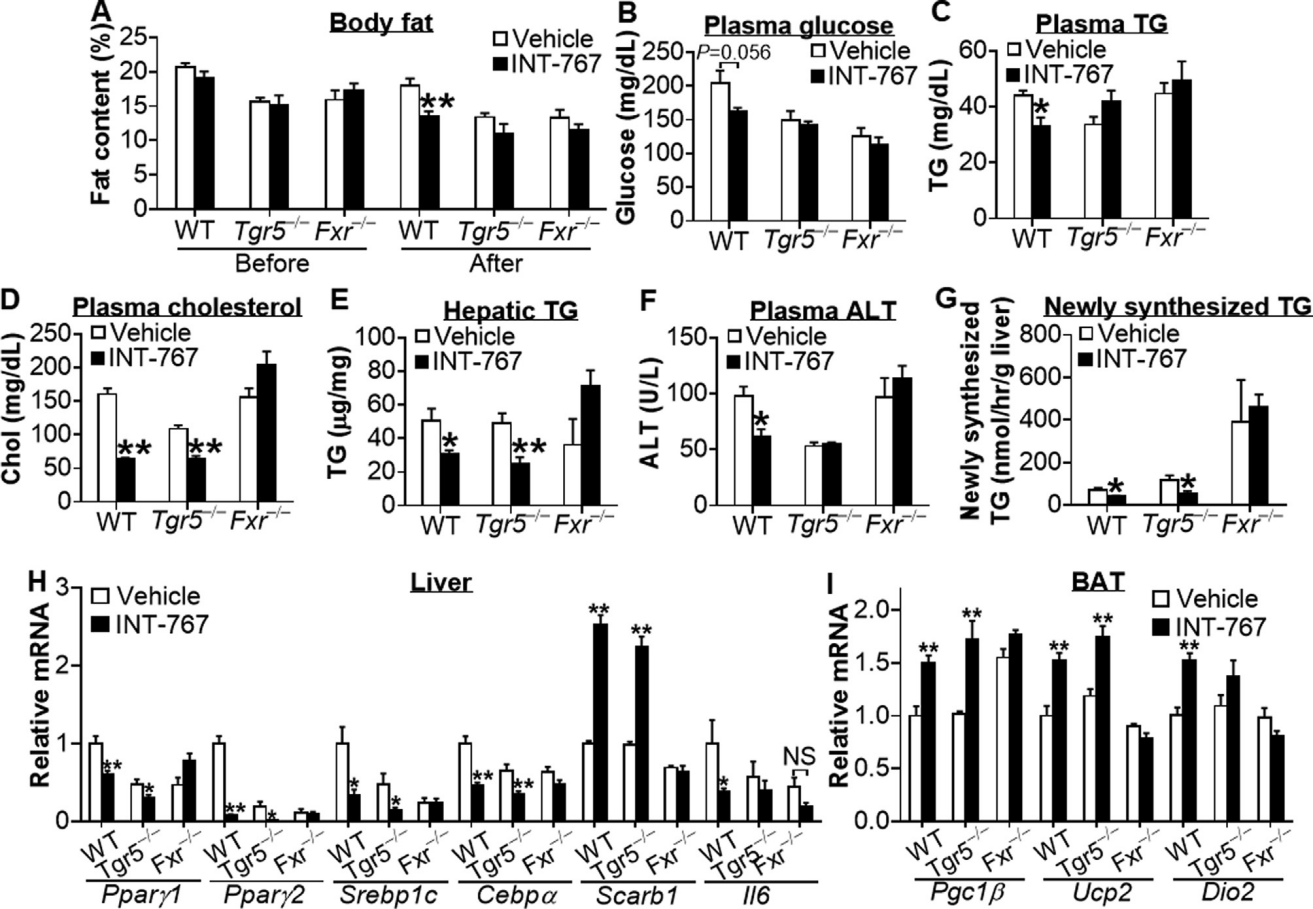
**INT-767 reverses HFD-induced metabolic disorders via activation of TGR5 and/or FXR.** Male WT mice, *Tgr5*^−/−^ mice and *Fxr*^−/−^ mice were fed a high fat diet (60% kcal from fat) for 12 weeks (n = 6–7 per group). By the end of week 12, mice were gavaged with either vehicle or INT-767 once a day for 10 days. (A) Body fat was measured by Echo-MRI before or after INT-767 treatment. (B) Plasma glucose. (C) Plasma TG. (D) Plasma cholesterol. (E) Hepatic TG. (F) Plasma ALT. (G) Hepatic *de novo* TG synthesis was analyzed by GC mass spectrometry. (H) Hepatic mRNA levels. (I) mRNA levels in brown adipose tissue (BAT). NS, not significant. *P < 0.05, **P < 0.01.

Prevention and treatment of diseases are both clinically desirable. So far, it is unclear whether activation of BA receptors can reverse diet-induced metabolic disorders. WT, *Tgr5*^−/−^ or *Fxr*^−/−^ mice were fed a high fat diet (HFD) for 12 weeks. By the end of week 12, mice were gavaged with either vehicle or INT-767 once a day for 10 days. While the baseline levels of body weight or body fat were similar between the vehicle and INT-767-treated groups (Supplementary Figure 1A and data not shown), INT-767 treatment significantly reduced body weight (Supplementary Figure 1B) and body fat content (Figure 2A) in WT mice but not in *Tgr5*^−/−^ or *Fxr*^−/−^ mice. INT-767 tended to reduce plasma glucose levels (Figure 2B) but significantly lowered plasma triglyceride (TG) levels (Figure 2C) in WT mice but not in in *Tgr5*^−/−^ or *Fxr*^−/−^ mice. In addition, INT-767 significantly reduced plasma cholesterol levels (Figure 2D) and hepatic TG levels (Figure 2E) in both WT mice and *Tgr5*^−/−^ mice but not in *Fxr*^−/−^ mice. Finally, INT-767 lowered plasma alanine aminotransferase (ALT) levels in wild-type mice but not in *Tgr5*^−/−^ or *Fxr*^−/−^ mice (Figure 2F). In contrast, INT-767 did not affect hepatic cholesterol levels, plasma aspartate aminotransferase (AST) levels, or plasma β-hydroxytutyrate (β-HB) levels (Supplementary Figure 1C–E). Plasma β-HB levels are an indicator of hepatic fatty acid oxidation (FAO). Our data suggest that INT-767 does not affect hepatic FAO.

Taken together, the data of Figure 2A–F suggest that INT-767 reverses HFD-induced obesity and liver dysfunction via activation of both FXR and TGR5, whereas the reversal of hypercholesterolemia or liver steatosis by INT-767 is dependent on activation of FXR. Thus, both FXR and TGR5 are involved in INT-767-mediated alleviation of metabolic disorders.

### 3.3 INT-767 regulates genes involved in lipogenesis, lipoprotein uptake, inflammation, and energy uncoupling in HFD-fed mice

To determine whether INT-767 regulated *de novo* lipogenesis, we injected mice with ^2^H_2_O. Our data show that INT-767 inhibited hepatic TG synthesis in both WT and *Tgr5*^−/−^ mice but not in *Fxr*^−/−^ mice (Figure 2G). In contrast, INT-767 did not affect hepatic very low-density lipoprotein (VLDL) secretion (Supplementary Figure 1F).

Consistent with the phenotypic or functional changes (Figure 2B–E,G), INT-767 treatment markedly reduced *Pparγ1*, *Pparγ2*, *Srebp1c*, and *Cebpα* but induced *SR-BI* by >2.5 fold in the livers of both WT and *Tgr5*^−/−^ mice but not *Fxr*^−/−^ mice (Figure 2H). Activation of FXR or TGR5 is known to repress inflammation by inhibiting NF-κB activity [1]. INT-767 repressed hepatic interleukin 6 (*Il6*) mRNA levels by 63% in WT mice but not in *Tgr5*^−/−^ or *Fxr*^−/−^ mice (Figure 2H), supporting the finding that INT-767 lowers plasma ALT levels dependent on activation of both TGR5 and FXR (Figure 2F).

In brown adipose tissue (BAT), INT-767 induced PPARγ coactivator 1β (*Pgc1β*), uncoupling protein 2 (*Ucp2*), and iodothyronine deiodinase 2 (*Dio2*) in WT mice, and the induction was absent both in *Tgr5*^−/−^ mice and in *Fxr*^−/−^ mice (Figure 2I). INT-767 did not have much effect on *Pgc1α*, *Ucp1*, or *Ucp3* expression (Supplementary Figure 1G). The reason for INT-767's failure to induce these latter genes is unclear at this time. Nonetheless, the data of Figure 2I support the finding that INT-767 reduces obesity via activation of both TGR5 and FXR (Figure 2A).

In addition to regulating genes involved in lipid or energy metabolism, INT-767 treatment also increased hepatic phosphoenolpyruvate carboxylase 1 (*Pck1*) and repressed glucose 6-phosphate (*G6pc*) in WT mice but not in *Tgr5*^−/−^ or *Fxr*^−/−^ mice (Supplementary Figure 1G). This observation is consistent with a previous finding that activation of FXR induces PCK1 but inhibits G6PC in the liver [28]. The induction of PCK1 but repression of G6PC by INT-767 may partly explain why INT-767 does not have a pronounced effect on plasma glucose levels (Figure 2B), glucose tolerance or insulin sensitivity (Supplementary Figure 2A–F).

### 3.4 INT-767 prevents the development of obesity by inducing energy expenditure in *Apoe*^−/−^ mice

**Figure 3:**
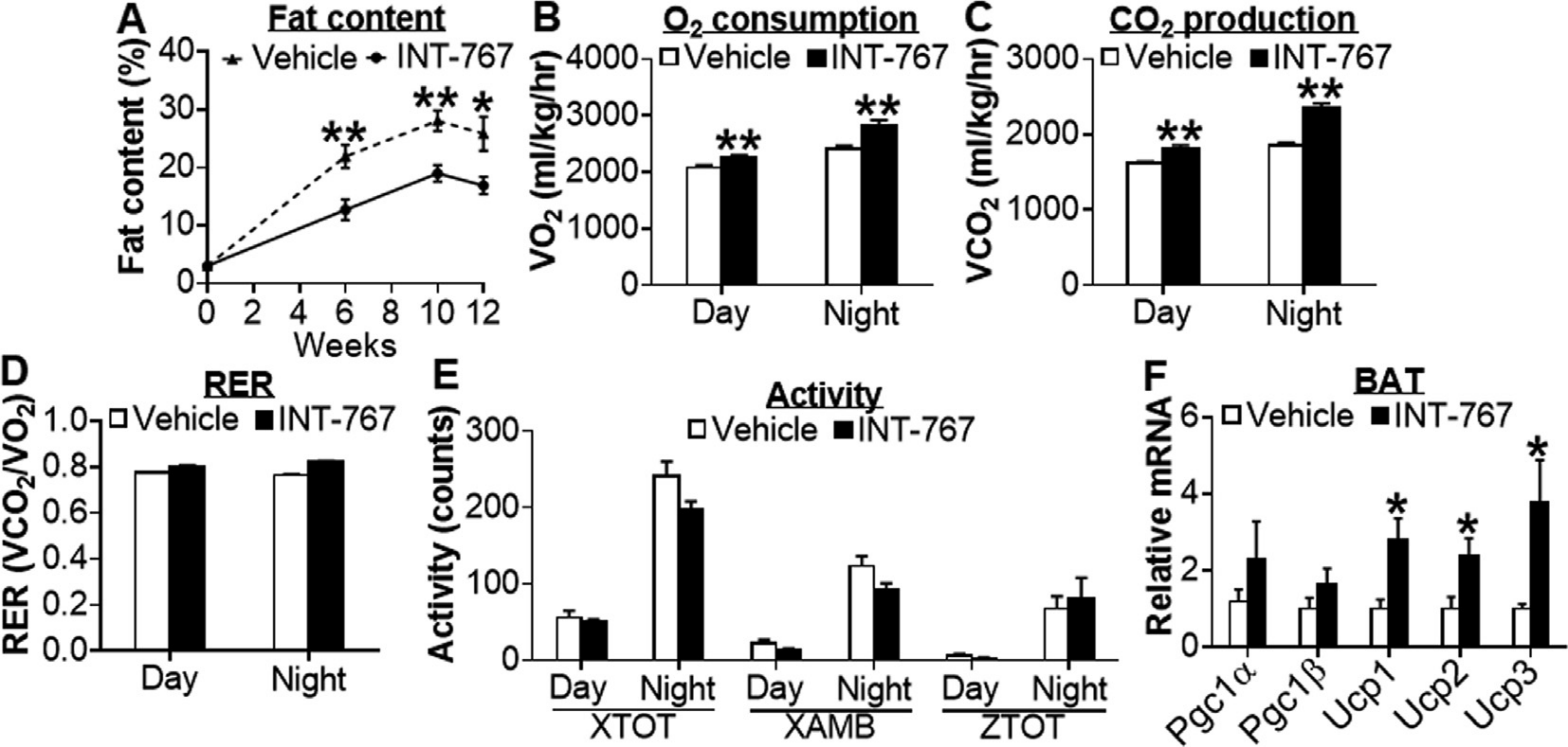
**INT-767 reduces obesity by increasing energy expenditure in *Apoe***^***−/−***^**mice.** Male *Apoe*^−/−^ mice were fed a Western diet (42% fat/0.2% cholesterol) for a total of 12 weeks (n = 7 per group). In the meanwhile, these mice were also gavaged with either vehicle or INT-767 (30 mg/kg, once a day). (A) Fat content. (B) Average O_2_ consumption during day or night time. (C) Average CO_2_ production during day or night time. (D) Respiratory exchange ratio (RER). (E) XTOT, XAMB or ZTOT activities during day or night time. (F) mRNA levels in BAT. *P < 0.05, **P < 0.01.

Next, we investigated whether long-term treatment with INT-767 can prevent metabolic disorders in *Apoe*^−/−^ mice, which can develop all the features of diet-induced metabolic disorders, including obesity, NAFLD, and atherosclerosis. *Apoe*^−/−^ mice were fed a Western diet for a total of 12 weeks. Meanwhile, these mice were also gavaged with either vehicle or INT-767 once a day. INT-767 treatment did not affect body weight or food intake (Supplementary Figure 3A and 3B). However, INT-767 reduced body fat content by 58%, 67%, and 65% at weeks 6, 10, and 12, respectively (Figure 3A). The reduction in body fat content was accompanied by an increase in lean mass (*P* < 0.05; data not shown). In addition, INT-767 treatment increased oxygen consumption (Figure 3B) and CO_2_ production (Figure 3C) during day or night time over a 24-h period (Supplementary Figure 3C and 3D) and induced energy expenditure (EE) during night time (data not shown). There was no change in respiratory exchange ratio (RER) (Figure 3D) or activities, including total beam breaks on the X-axis (XTOT), ambulatory activity on the X-axis (XAMB), and total beam breaks on the Z-axis (ZTOT) (Figure 3E). In BAT, *Ucp1*, *Ucp2*, and *Ucp3* mRNA levels were significantly induced (Figure 3F). Together, the data of Figure 3 demonstrate that long-term treatment with INT-767 prevents the development of obesity by inducing energy expenditure in *Apoe*^−/−^ mice.

### 3.5 INT-767 prevents the progression of atherosclerosis and NAFLD in *Apoe*^−/−^ mice

In addition to preventing the development of obesity, INT-767 treatment also reduced plasma TG and cholesterol levels by ∼50% (Supplementary Figure 4A–C), but had no effect on plasma glucose levels (Supplementary Figure 4D). Analysis of plasma lipoprotein profile by FPLC indicated that INT-767 reduced circulating levels of VLDL-cholesterol and VLDL-TG (Supplementary Figure 4E and 4F). In agreement with the marked reduction in plasma lipoproteins, atherosclerotic lesions in both aortas and aortic roots were reduced by >50% (Supplementary Figure 4G–J). These data demonstrate that INT-767 improves hyperlipidemia and protects from the progression of atherosclerosis in *Apoe*^−/−^ mice.

In the liver, treatment with INT-767 reduced hepatic levels of total cholesterol and TG (Supplementary Figure 5A and 5B). Oil Red O staining of liver sections indicated that INT-767 reduced neutral lipid accumulation (Supplementary Figure 5C). Consistent with the improved lipid homeostasis in *Apoe*^−/−^ mice, INT-767 treatment significantly inhibited *Pparγ1, Pparγ2, Srebp1c*, and *Cebpα* but induced *SR-BI* expression (Supplementary Figure 5D). In addition, INT-767 inhibited the expression of inflammatory cytokines *Tnfα* and *Il1β*, and genes involved in fibrogenesis, including tissue inhibitor of metalloproteinase 1 (*Timp1*), alpha smooth muscle actin (*α-Sma*), transforming growth factor β (*Tgfβ*), and collagen type 1 α1 (*Col1α1*) (Supplementary Figure 5E). In agreement with the changes in gene expression, INT-767 treatment alleviated fibrosis in the liver of *Apoe*^−/−^ mice (Supplementary Figure 5F).

### 3.6 INT-767 promotes regression of atherosclerosis in *Apoe*^−/−^ mice

**Figure 4:**
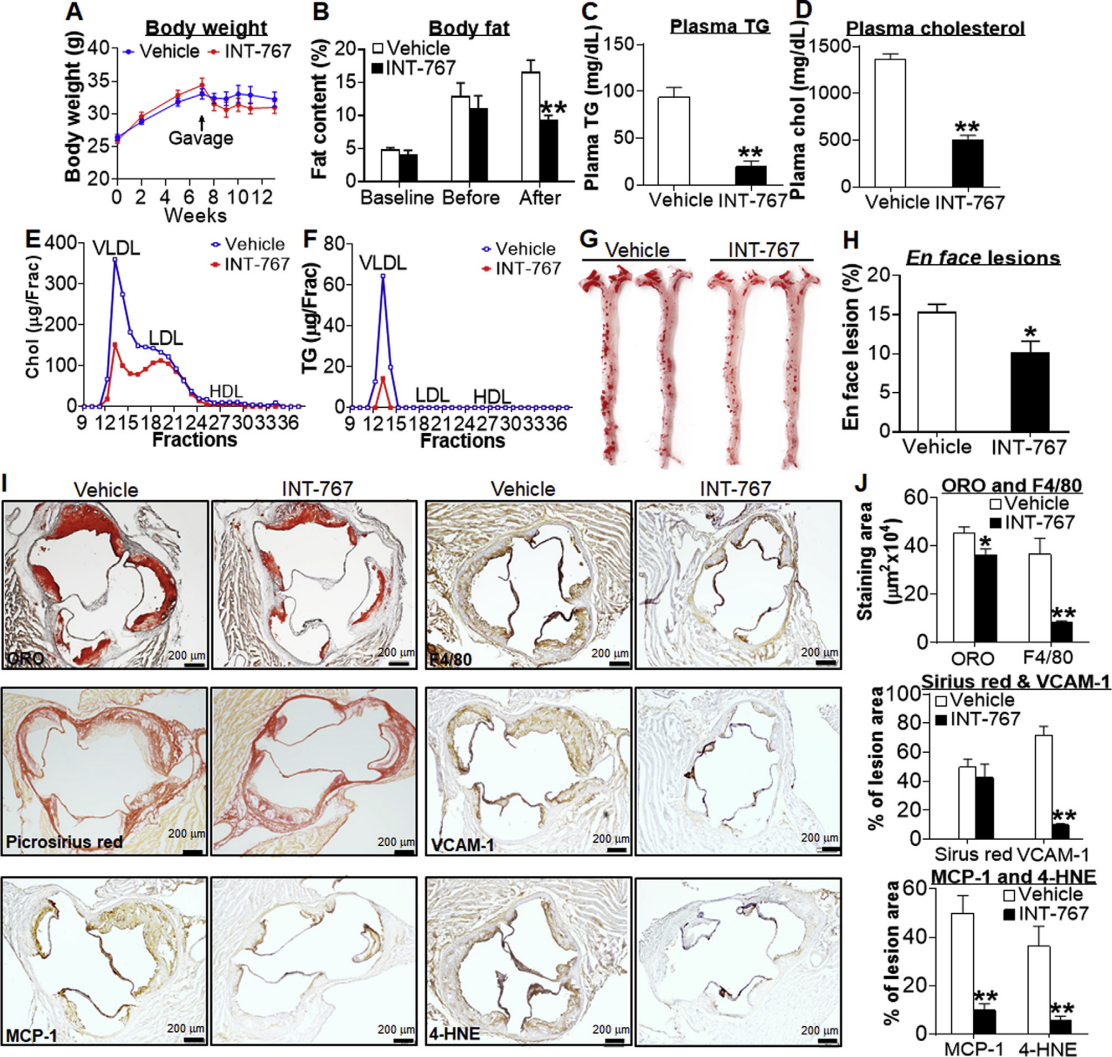
**INT-767 promotes regression of diet-induced hyperlipidemia and atherosclerosis in *Apoe***^**−/−**^**mice.** Male *Apoe*^−/−^ mice were fed a Western diet (42% fat/0.2 cholesterol) for 7 weeks, and then gavaged with either vehicle or INT-767 (30 mg/kg, once a day) for 6 weeks (n = 8 per group). (A) Body weight. (B) Body fat content at baseline, before, or after gavage with INT-767. (C) Plasma TG levels. (D) Plasma total cholesterol levels. (E) Circulating cholesterol lipoprotein distribution. Chol, cholesterol. (F) Circulating TG lipoprotein distribution. (G) Representative *en face* aortas stained with Oil Red O (ORO). (H) Average lesion size of *en face* aortas. (I) Representative aortic root sections stained with ORO (left top panel) or picrosirius red (left middle panel), or immunostained for detection of MCP-1 (left bottom panel), F4/80 (right top panel), VCAM-1 (right middle panel) or 4-HNE (right bottom panel). (J) Quantification of average staining areas per section shown in (I). *P < 0.05, **P < 0.01.

To determine whether INT-767 was able to reverse atherosclerosis, we fed *Apoe*^−/−^ mice a Western diet for 7 weeks. These mice were then gavaged with either vehicle or INT-767 for 6 weeks. There was no significant change in body weight before or after INT-767 treatment (Figure 4A). However, INT-767 reduced fat content by 44% (Figure 4B) and increased lean mass content (*P* < 0.05; data not shown). INT-767 also markedly lowered plasma TG levels by 80% (Figure 4C) and plasma cholesterol levels by 64% (Figure 4D). The reduction in plasma cholesterol levels was mainly due to a reduction in VLDL-C and LDL-C (Figure 4E), whereas the reduction in plasma TG levels was due to a reduction in VLDL-TG (Figure 4F). Consistent with the marked reduction in plasma TG and cholesterol levels, INT-767 decreased atherosclerotic lesions in both the aortas (Figure 4G,H) and aortic roots [Figure 4I (top left panel) and 4J]. In addition, in the atherosclerotic lesions there was a marked reduction in protein levels of F4/80, monocyte chemoattractant protein 1 (MCP-1/CCL2), vascular cell adhesion molecule 1 (VCAM-1) and 4-hydroxynonenal (4-HNE) (Figure 4I,J), suggesting that INT-767 inhibits monocyte/macrophage infiltration and oxidative stress in vasculature. In contrast, there was no significant reduction in collagen accumulation in the lesions (Figure 4I,J). These data demonstrate that INT-767 improves hyperlipidemia and promotes regression of atherosclerosis in *Apoe*^−/−^ mice.

### 3.7 INT-767 promotes regression of NAFLD in *Apoe*^−/−^ mice

**Figure 5:**
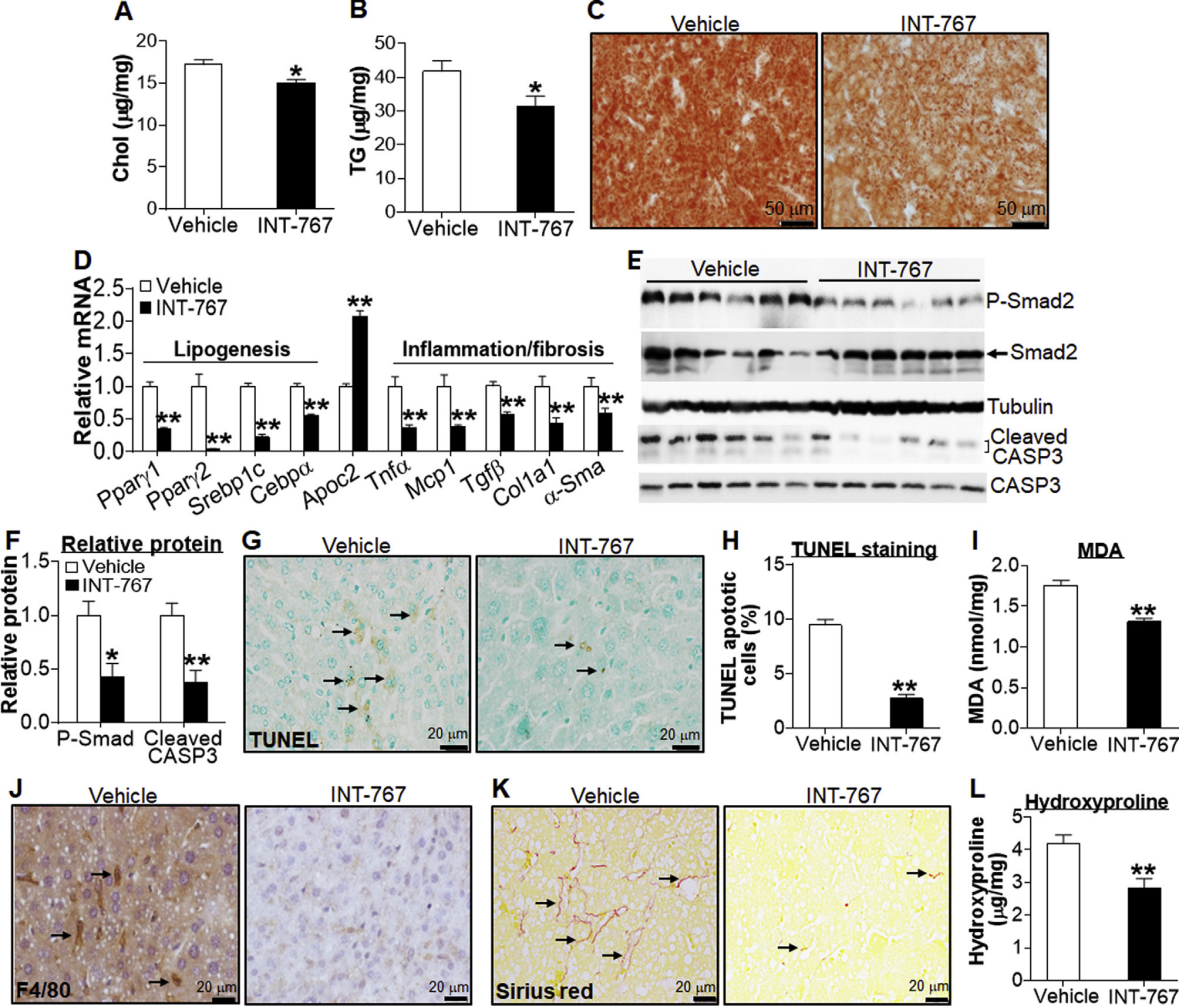
**INT-767 promotes regression of NAFLD in *Apoe***^**−/−**^**mice.** Male *Apoe*^−/−^ mice have been described in the legend of Figure 4 (n = 8 per group). (A) Hepatic total cholesterol levels. (B) Hepatic TG levels. (C) Representative liver section images stained by Oil Red O. (D) Hepatic mRNA levels. (E and F) Hepatic proteins were detected by immunoblots (E) and then quantified (F). (G) Detection of apoptotic cells by TUNEL assays. (H) Quantification of apoptotic cells (%). (I) Hepatic MDA levels. (J) Liver sections were immunostained for detection of F4/80. (K) Representative liver images stained by Picrosirius red. (L) Hepatic hydroxyproline levels. Arrows point to staining-positive cells (G, J) or fibrosis (K). *P < 0.05, **P < 0.01.

Western diet-fed *Apoe*^−/−^ mice are prone to develop both atherosclerosis and NAFLD. In the liver, INT-767 significantly reduced the accumulation of cholesterol and TG in Western diet-fed *Apoe*^−/−^ mice (Figure 5A–C). Consistent with the reduced hepatic TG accumulation, hepatic *Pparγ1*, *Pparγ2*, *Srebp1c*, and *Cebpα* were reduced by >50% (Figure 5D). INT-767 also induced hepatic apolipoprotein C2 (*Apoc2*) by 2-fold (Figure 5D). ApoC-II is a co-activator of lipoprotein lipase (LPL) and is known to be induced by FXR [9]. Thus, INT-767 reduces plasma VLDL levels (see Figure 4) likely via induction of ApoC-II and subsequent activation of LPL for TG hydrolysis in the plasma. In addition, INT-767 markedly inhibited the expression of a number of inflammatory or fibrogenic genes, such as *Tnfα*, *Mcp1/Ccl2*, *Tgfβ*, *Col1a1*, and α*-Sma* (Figure 5D), and reduced protein levels of phospho-Smad2 and Caspase 3 (Figure 5E,F).

Consistent with the changes in gene expression (Figure 5D–F), INT-767 reduced hepatic levels of apoptosis (Figure 5G,H), malondialdehyde (MDA) (Figure 5I), macrophage infiltration (Figure 5J. *P* < 0.01), and collagen accumulation, as determined by picrosirius red staining (Figure 5K) and hydroxyproline quantitation (Figure 5L). Together, the data of Figure 5 demonstrate that INT-767 promotes regression of NAFLD in *Apoe*^*−/−*^ mice.

In addition, we also determined whether INT-767 improved energy expenditure in the regression mouse model. Our data show that INT-767 increased O_2_ consumption and CO_2_ production during the night time without affecting RER or activities but induced *Ucp2* and *Dio2* expression in BAT (Supplementary Figure 6A–E). These data, together with the data of Figure 3, demonstrate that INT-767 induces energy expenditure in both the progression and regression models of atherosclerosis.

### 3.8 FXR inhibits lipogenic genes via both SHP-dependent and SHP-independent pathways

**Figure 6:**
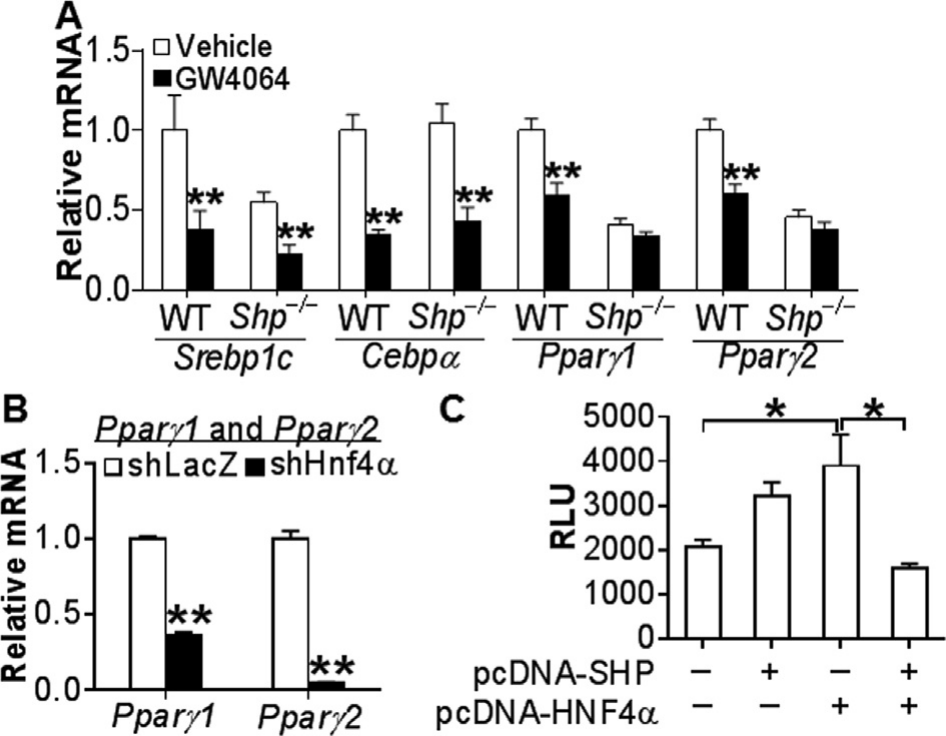
**Activation of FXR inhibits hepatic lipogenic genes via SHP-dependent and SHP-independent pathways.** (A) Chow-fed male *Shp*^+/+^ and *Shp*^−/−^ mice were gavaged with either vehicle or GW4064 (30 mg/kg; twice a day) for 7 days (n = 8 mice per group). Hepatic mRNA levels were quantified by qRT-PCR. (B) Chow-fed male C57BL/6 mice were injected i.v. with Ad-shLacZ or Ad-shHnf4α (n = 7 per group). After 7 days, hepatic *Pparγ1* and *Pparγ2* mRNA levels were determined. (C) *Pparγ2* luciferase-promoter plasmid was co-transfected with SHP and/or HNF4α expression plasmids. After 36 h, relative luciferase units (RLU) were determined. *P < 0.05, **P < 0.01.

Our data have shown that INT-767 regulates hepatic lipogenesis and TG levels via activation of FXR. Small heterodimer partner (SHP) is an atypical nuclear receptor that does not have a DNA binding domain, but modulates gene expression by interacting with other transcription factors [38]. Activation of FXR is known to induce SHP expression [9]. GW4064 is a specific FXR agonist and is shown to regulate lipid metabolism via activation of FXR [37]. In order to investigate the role of SHP in FXR-mediated repression of lipogenic genes, we treated WT mice and *Shp*^−/−^ mice with either vehicle or GW4064 for 7 days. GW4064 inhibited *Srebp1c* and *Cebpα* in both WT mice and *Shp*^−/−^ mice (Figure 6A). In contrast, GW4064 inhibited *Pparγ1* and *Pparγ2* in WT mice but not in *Shp*^−/−^ mice (Figure 6A), indicating that inhibition of PPARγ by FXR requires SHP.

We then investigated how SHP inhibits PPARγ expression. SHP has been shown to interact with hepatocyte nuclear factor 4α (HNF4α) to inhibit gene expression [39]. In mice deficient in hepatic *Hnf4α*, both *Pparγ1* and *Pparγ2* mRNA levels were markedly reduced (Figure 6B). In transient transfection assays, HNF4α over-expression induced *Pparγ* promoter activity and this induction was repressed by SHP over-expression (Figure 6C), suggesting that SHP inhibits PPARγ promoter activity likely through HNF4α.

## 4 Discussion

Activation of FXR or TGR5 has overlapping but also differing effects on metabolic regulation and inflammation [1]. In this report, we demonstrate that in addition to preventing the progression of obesity, NAFLD, and atherosclerosis, activation of BA receptors can also promote regression of these common metabolic disorders. Mechanistically, we demonstrate that BA receptor activation reduces obesity, induces thermogenic genes and inhibits inflammatory response via activation of FXR and TGR5. We also demonstrate that bile acid receptor activation inhibits hepatic lipogenic genes and lipogenesis via activation of FXR. Given that long-term activation of FXR is reported to induce obesity [17] and that activation of TGR5 alone has a limited effect on lipid metabolism, our data suggest that targeting both FXR and TGR5 is a more attractive strategy for treatment of common metabolic disorders, such as obesity, NAFLD, and atherosclerosis.

NAFLD is one of the most common liver diseases worldwide and is also an independent risk factor for cardiovascular diseases. Our data clearly show that bile acid receptor activation reduces lipogenesis and liver steatosis via activation of FXR. Although FXR activation has been shown to reduce hepatic TG levels, the underlying mechanism is not well understood [1], [40]. FXR is shown to inhibit hepatic SREBP-1c expression, but FXR does not inhibit SREBP-1c downstream target genes [1], [40], suggesting that the FXR-SREBP-1c pathway is not critical. Previously, we have shown that the induction of hepatic carboxylesterase 1 may partly account for the reduction in hepatic TG levels following FXR activation [25]. In the present study, we show that both INT-767 and specific activation of FXR by GW4064 repress several genes involved in lipogenesis in both chow- and HFD-fed mice. In addition to SREBP-1c we identify PPARγ and CEBPα as novel FXR-regulated genes. We further show that FXR inhibits PPARγ expression by a mechanism requiring SHP whereas the inhibition of CEBPα or SREBP-1c by FXR is independent of SHP. Importantly, we demonstrate that INT-767 inhibits hepatic lipogenesis via activation of FXR. These novel data, together with previous observations [1], [40], indicate that activation of FXR inhibits lipogenesis likely via repressing PPARγ and/or CEBPα. The finding that INT-767 does not affect VLDL secretion or FAO (as determined by unchanged plasma β-HB levels) suggests that INT-767 improves liver steatosis likely via inhibition of hepatic lipogenesis.

We have previously shown that activation of FXR induces hepatic SR-BI expression and increases reverse cholesterol transport [37], [41]. FXR activation is also reported to inhibit platelet activation *in vitro* [42]. Consistent with these observations, INT-767 induces hepatic SR-BI expression and inhibits or reverses the development of atherosclerosis in *Apoe*^−/−^ mice. INT-767 markedly reduces VLDL-TG levels in *Apoe*^−/−^ mice, at least partly due to induction of ApoC-II. Other mechanism(s) may also be involved in the hypolipidemic effect of INT-767. For instance, INT-767 may inhibit fat absorption via suppressing bile acid production. On the other hand, TGR5 activation has been shown to inhibit macrophage inflammation [16]. Indeed, we find that INT-767 inhibits monocyte/macrophage infiltration and oxidative stress in atherosclerotic lesions. Thus, INT-767 inhibits or reverses the development of atherosclerosis likely by improving hyperlipidemia, promoting RCT, and suppressing macrophage inflammation.

In addition to regulating lipid homeostasis, our data show that INT-767 reduces obesity and improves energy expenditure. Previous studies show that activation of TGR5 improves energy homeostasis by inducing thermogenic genes in BAT [15]. In contrast, activation of FXR is shown to increase diet-induced obesity [17] whereas loss of FXR prevents diet-induced or genetic obesity [30]. Interestingly, our data suggest that INT-767 reduces obesity via activation of both TGR5 and FXR. Nonetheless, we realize that both *Fxr*^−/−^ mice [30] and *Tgr5*^−/−^ mice [43] are resistant to diet-induced obesity to some degrees, which makes the use of these two animal models imperfect for interpreting the results. It is unclear why FXR activation is also involved in INT-767-mediated alleviation of obesity. One possibility is that FXR activation is known to induce hepatic expression of FGF21 [44], a well-characterized inducer of thermogenesis. Despite its anti-obesity effect, INT-767 fails to reverse diet-induced glucose intolerance or insulin insensitivity, suggesting that INT-767 may not be useful for treatment of type II diabetes.

Activation of FXR or TGR5 inhibits inflammatory response via repressing NF-κB activity [1]. FXR is not expressed or expressed at a very low level in macrophages [45] (data not shown), but highly expressed in hepatocytes. In contrast, TGR5 is expressed in macrophages but not in hepatocytes. Our data suggest that both FXR and TGR5 play a role in INT-767-mediated inhibition of hepatic inflammatory cytokines, consistent with the anti-inflammatory function of both FXR and TGR5.

NAFLD, obesity and atherosclerosis are common metabolic disorders. So far, there are very limited approaches/treatments that can simultaneously ameliorate these metabolic disorders. By using several mouse models, we show that BA receptor activation can prevent or regress these metabolic disorders and that these effects are mediated by activation of FXR and/or TGR5. Together, our data suggest that simultaneously targeting both FXR and TGR5 may be a more attractive approach for treatment of common metabolic disorders than individually targeting FXR or TGR5.

## Appendix A Supplementary data

mmc1
