# Supplementary material for: Reversal of metabolic disorders by pharmacological activation of bile acid receptors TGR5 and FXR
Source: Mol Metab. 2018 Jan 11;9:131–40. doi: 10.1016/j.molmet.2018.01.005 (PMC5870099; doi:10.1016/j.molmet.2018.01.005)

Supplementary Materials

Reversal of metabolic disorders by pharmacological activation of bile acid receptors FXR and TGR5

Kavita Jadhav [1*], Yang Xu [1*], Yanyong Xu [1*], Yuanyuan Li [1], , Jiesi Xu [1,2], Yingdong Zhu [1], Huihui Sun [1], Luciano Adorini [3], Yoon Kwang Lee [1], Takhar Kasumov [4], Liya Yin [1] and Yanqiao Zhang [1**]

[1]Department of Integrative Medical Sciences, Northeast Ohio Medical University, Rootstown, OH 44272, USA

[2]State Key Laboratory of Molecular Developmental Biology, Institute of Genetics and Developmental Biology, Chinese Academy of Sciences, Beijing 100101, China

[3]Intercept Pharmaceuticals, New York, NY 10014, USA

[4]Department of Pharmaceutical Sciences, Northeast Ohio Medical University, Rootstown, OH 44272, USA

* Contribute equally to the work.

Running title: FXR or TGR5-dependent metabolic regulation

Corresponding author:
Yanqiao Zhang, MD
Department of Integrative Medical Sciences
Northeast Ohio Medical University
Phone: (330) 325-6693
Fax: (330) 325-5978
Email: yzhang@neomed.edu

FIGURE LEGENDS

Figure 1: Metabolic effect of INT-767 in HFD-fed wild-type mice, *Tgr5*$^{-/-}$ mice or *Fxr*$^{-/-}$ mice. Male wild-type (WT) mice, *Tgr5*$^{-/-}$ mice or *Fxr*$^{-/-}$ mice were fed a high fat diet (60% kcal from fat) for 12 weeks (n=6-7). At the end of week 12, mice were gavaged with either vehicle or INT-767 once a day for 10 days. (A) Body fat content at the baseline levels. (B) Body weight before or after gavage with INT-767. (C) Hepatic cholesterol. (D) Plasma AST. (E) Plasma β-hydroxybutyrate levels. (F) VLDL secretion was performed in male WT mice after mice were fasted for 16 h and then injected i.v. with Tyloxapol (500 mg/kg) (n=8). (G) Hepatic mRNA levels. NS, not significant. * $P<0.05$, ** $P<0.01$

Figure 2: INT-767 treatment does not affect glucose tolerance or insulin sensitivity. Male wild-type (WT) mice, *Tgr5*$^{-/-}$ mice or *Fxr*$^{-/-}$ mice were fed a high fat diet (60% kcal from fat) for 12 weeks (n=6-7). At the end of week 12, mice were gavaged with either vehicle or INT-767 once a day for 10 days. (A-C) Glucose tolerance test (GTT) was performed in WT mice (A), *Tgr5*$^{-/-}$ mice (B) or *Fxr*$^{-/-}$ mice (C). (D-F) Insulin tolerance test (ITT) was performed in WT mice (D), *Tgr5*$^{-/-}$ mice (E) or *Fxr*$^{-/-}$ mice (F).

Figure 3: Effect of INT-767 treatment on energy expenditure in *Apoe*$^{-/-}$ mice. Male *Apoe*$^{-/-}$ mice were fed a Western diet (42% fat/0.2% cholesterol) for a total of 12 weeks (n=7). In the meanwhile, these mice were also gavaged with either vehicle or INT-767 (30 mg/kg, once a day). (A) Body weight. (B) Food intake. (C) 24-h VO_2 during the day or night time. (D) 24-h VCO_2 during the day or night time.

Figure 4: INT-767 markedly improves hyperlipidemia and protects against the development of atherosclerosis in *Apoe*$^{-/-}$ mice. Male *Apoe*$^{-/-}$ mice were fed a Western diet (42% fat/0.2% cholesterol) for a total of 12 weeks (n=7). (A) Representative plasma images. (B) Plasma TG levels. (C) Plasma total cholesterol levels. (D) Plasma glucose levels. (E) Circulating cholesterol lipoprotein distribution. (F) Circulating TG lipoprotein distribution. (G) Representative *en face* aortas stained with Oil Red O. (H) Average lesion size of *en face* aortas. (I) Representative aortic root sections stained with Oil Red O. (J) Average lesion size of aortic roots per section. * $P<0.05$, ** $P<0.01$

Figure 5: INT-767 inhibits the development of NAFLD in *Apoe*$^{-/-}$ mice. Male *Apoe*$^{-/-}$ mice were fed a Western diet (42% fat/0.2% cholesterol) for a total of 12 weeks (n=7). (A) Hepatic total cholesterol levels. (B) Hepatic TG levels. (C) Representative liver sections stained by Oil Red O. (D) Hepatic mRNA levels of genes involved in lipid metabolism. (E) Hepatic mRNA levels of genes involved in inflammation and fibrogenesis. (F) Representative liver sections stained by picrosirius red. In (F), arrows point to fibrosis. * $P<0.05$, ** $P<0.01$

Figure 6: INT-767 induces energy expenditure in *Apoe*$^{-/-}$ mice (reversal model). Male *Apoe*$^{-/-}$ mice were fed a Western diet (42% fat/0.2 cholesterol) for 7 weeks, and then gavaged with either vehicle or INT-767 (30 mg/kg, once a day) for 6 weeks (n=8 per group). (A) O_2 consumption. (B) CO_2 production. (C) Respiratory exchange ratio. (D) XTOT, XAMB and ZTOT activities. (E) mRNA levels in brown adipose tissue. * $P<0.05$

Supplementary Figure 1

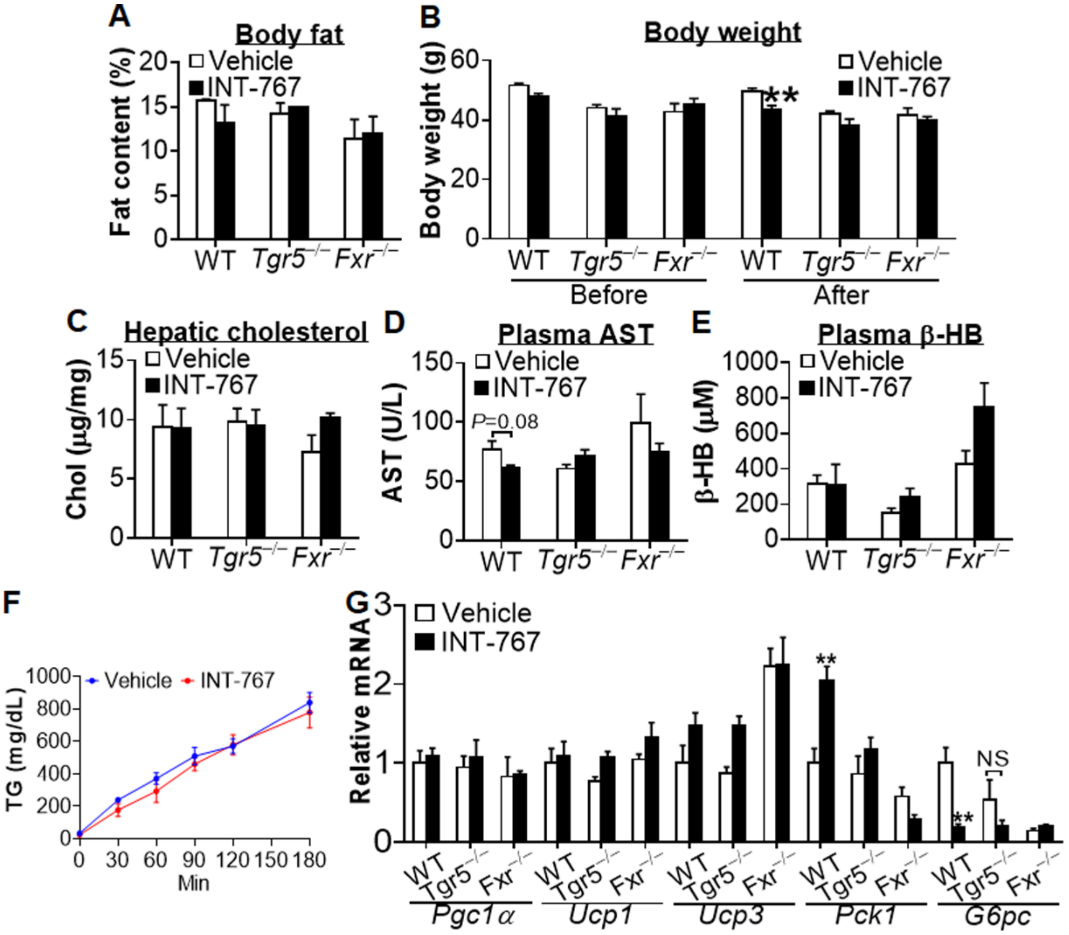

Supplementary Figure 2

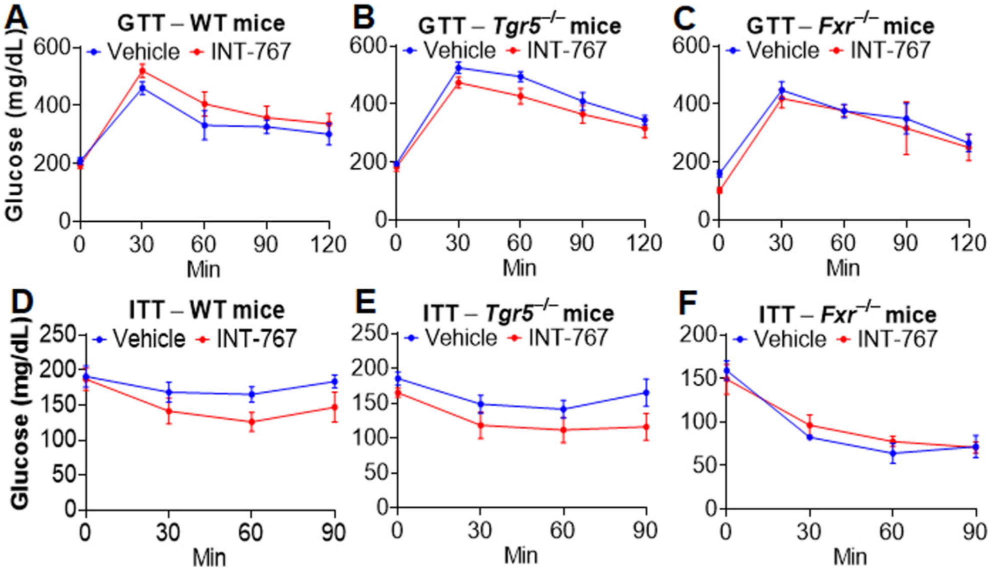

Supplementary Figure 3

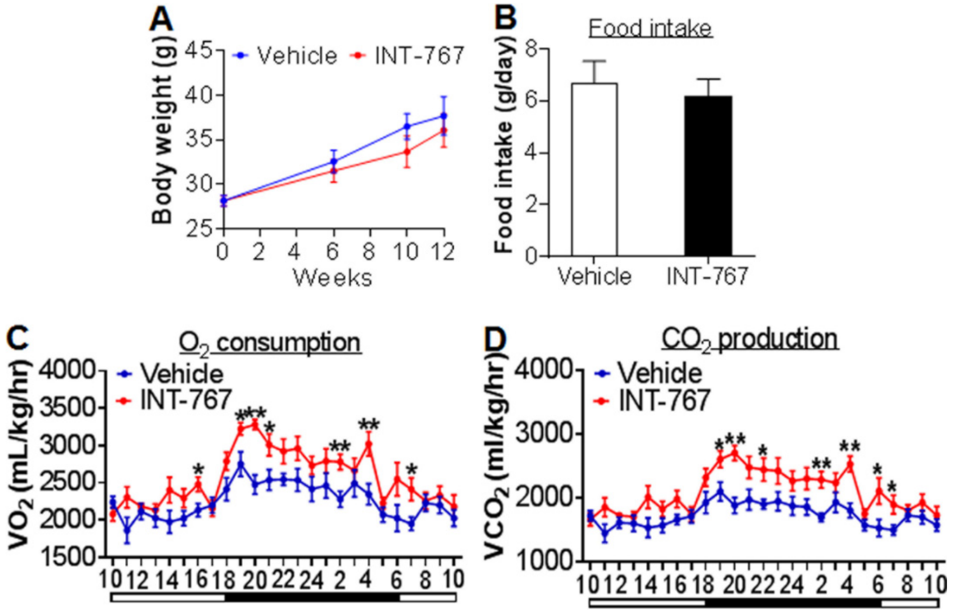

Supplementary Figure 4

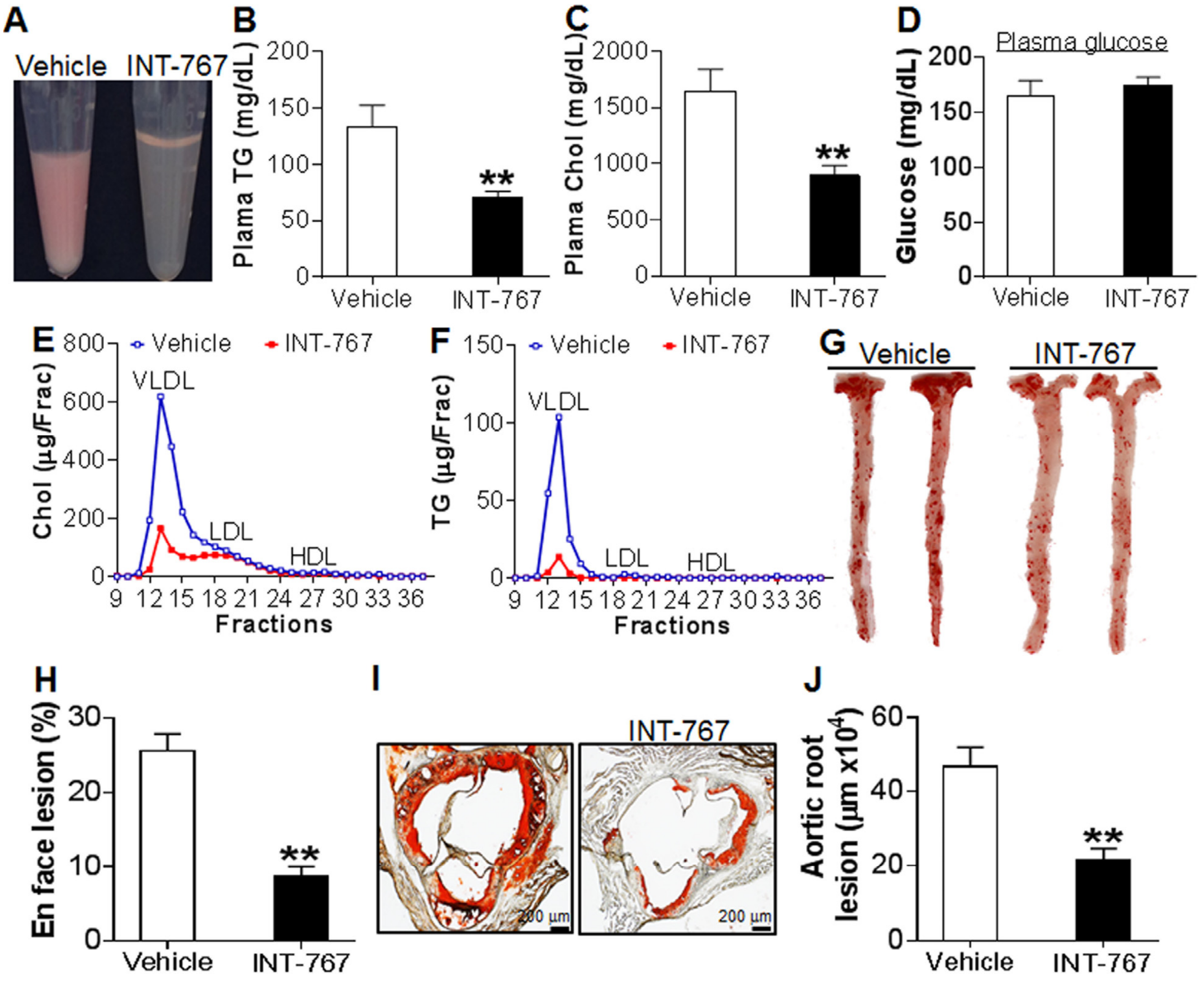

Supplementary Figure 5

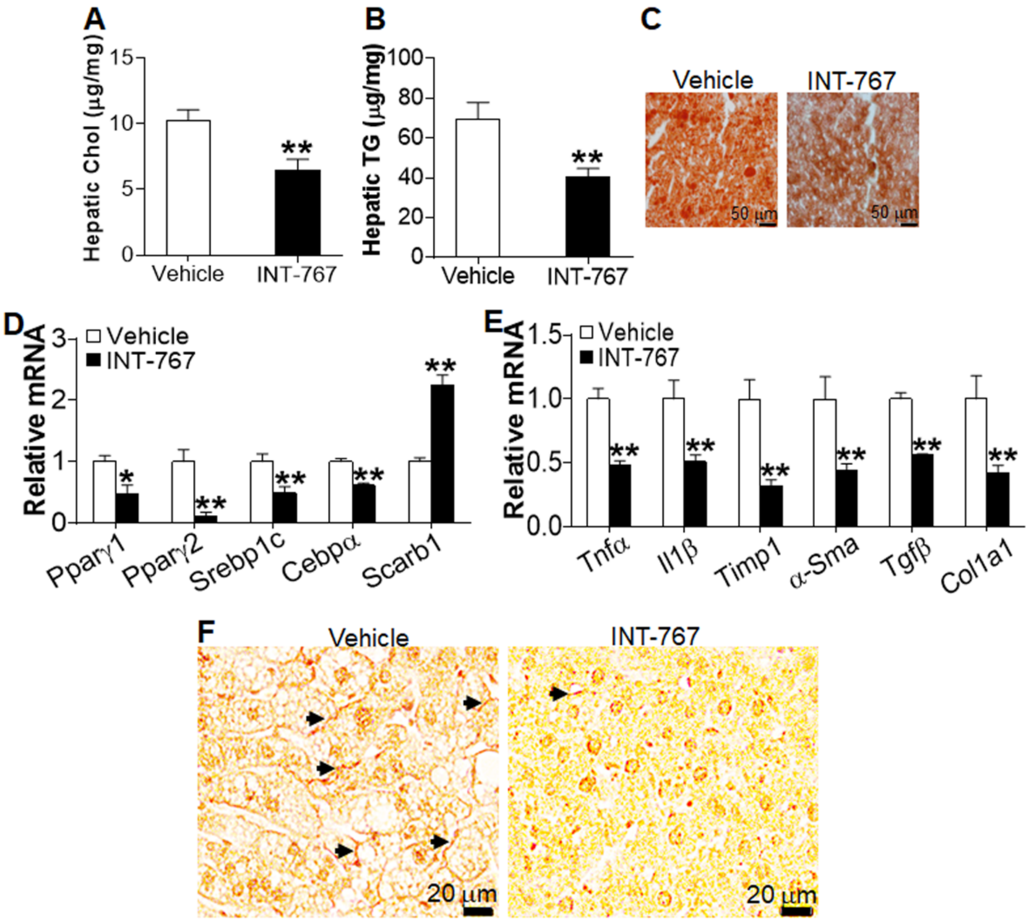

Supplementary Figure 6

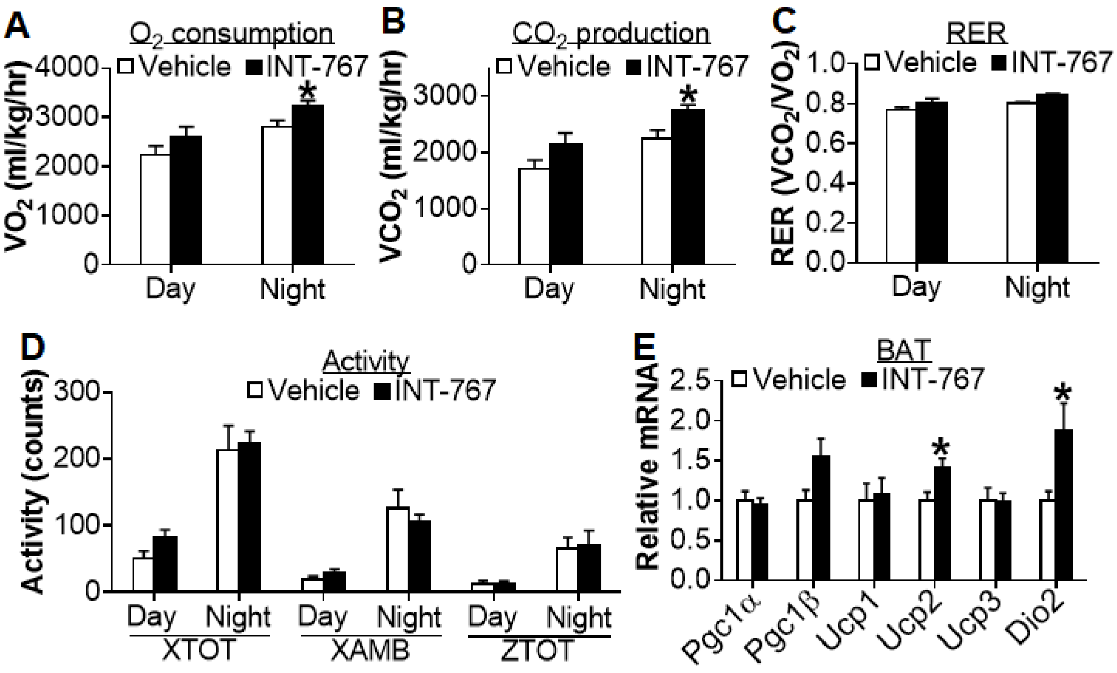

Supplement: mmc1 [file mmc1.pdf]
